# Associations of Amylin with Inflammatory Markers and Metabolic Syndrome in Apparently Healthy Chinese

**DOI:** 10.1371/journal.pone.0024815

**Published:** 2011-09-15

**Authors:** Xinwei Hou, Liang Sun, Zongmeng Li, Haiwei Mou, Zhijie Yu, Huaixing Li, Peizhen Jiang, Danxia Yu, Hongyu Wu, Xingwang Ye, Xu Lin, Yingying Le

**Affiliations:** 1 Key Laboratory of Nutrition and Metabolism, Institute for Nutritional Sciences, Shanghai Institutes for Biological Sciences, Chinese Academy of Sciences, Graduate School of the Chinese Academy of Sciences, Shanghai, China; 2 Shanghai Municipal Center for Disease Control & Prevention, Shanghai, China; Brigham & Women's Hospital, and Harvard Medical School, United States of America

## Abstract

**Background:**

Cellular and animal studies implicate multiple roles of amylin in regulating insulin action, glucose and lipid metabolisms. However, the role of amylin in obesity related metabolic disorders has not been thoroughly investigated in humans. Therefore, we aimed to evaluate the distribution of circulating amylin and its association with metabolic syndrome (MetS) and explore if this association is influenced by obesity, inflammatory markers or insulin resistance in apparently healthy Chinese.

**Methods:**

A population-based sample of 1,011 Chinese men and women aged 35–54 years was employed to measure plasma amylin, inflammatory markers (C-reactive protein [CRP] and interleukin-6 [IL-6]), insulin, glucose and lipid profiles. MetS was defined according to the updated National Cholesterol Education Program Adult Treatment Panel III criteria for Asian-Americans.

**Results:**

Plasma amylin concentrations were higher in overweight/obese participants than normal-weight counterparts (*P*<0.001) without sex difference. Circulating amylin was positively associated with CRP, IL-6, BMI, waist circumference, blood pressure, fasting glucose, insulin, amylin/insulin ratio, HOMA-IR, LDL cholesterol and triglycerides, while negatively associated with HDL cholesterol (all *P*<0.001). After multiple adjustments, the risk of MetS was significantly higher (odds ratio 3.71; 95% confidence interval: 2.53 to 5.46) comparing the highest with the lowest amylin quartile. The association remained significant even further controlling for BMI, inflammatory markers, insulin or HOMA-IR.

**Conclusions:**

Our study suggests that amylin is strongly associated with inflammatory markers and MetS. The amylin-MetS association is independent of established risk factors of MetS, including obesity, inflammatory markers and insulin resistance. The causal role of hyperamylinemia in the development of MetS needs to be confirmed prospectively.

## Introduction

Amylin, also named islet amyloid polypeptide (IAPP), is a 37 amino acid polypeptide initially isolated from amyloid deposit in pancreatic islets of type 2 diabetes patients [1], [2]. Amylin is co-secreted with insulin by β-cells in response to stimulation of glucose, free fatty acids and food intake [3]–[5]. Amylin is an important player in the control of energy balance [6]. Amylin inhibits food intake by promoting meal-ending satiation, possibly through stimulation of its receptor in the area postrema [7]. The nucleus of the solitary tract and the lateral parabrachial nucleus seem to be also involved in the inhibitory effect of amylin on food intake [6]. Amylin increases energy expenditure after peripheral and central administration in animals [6]. Chronic infusion of amylin into the brain reduces body weight gain and adiposity, while chronic infusion of an amylin receptor antagonist into the brain increases body adiposity [6], [8]. Amylin was found *in vitro* to inhibit both basal and insulin stimulated glycogenesis in rat skeleton muscle [9] and also to impair glucose disposal in liver cells [10]. Moreover, an animal study also showed that amylin could suppress insulin secretion by enhancing β-cell apoptosis, which consequently induced hepatic and extrahepatic insulin resistance and glucose dysregulation in human IAPP transgenic rats [11]. Infusion of amylin in dog also induced peripheral insulin resistance [12]. Elevated levels of amylin were observed in obese children [13] and obese adults with impaired glucose tolerance or type 2 diabetes [14], or in women with polycystic ovary syndrome and preterm neonates with feed intolerance [15], [16]. However, the pathophysiological role(s) of amylin in human metabolic diseases have not been studied in large-scale populations.

Metabolic syndrome (MetS), an aggregation of metabolic abnormalities comprising central obesity, dyslipidemia, elevated blood pressure and hyperglycemia, is a generally acknowledged precursor of type 2 diabetes and cardiovascular disease (CVD) [17]. A recent meta-analysis showed that MetS is associated with a 2-fold increase in risk of CVD, CVD mortality, and stroke [18]. Owing to excess caloric intake and a sedentary lifestyle, global epidemic trends of obesity and MetS have become one of the public health challenges not only in Western countries [19] but also in Asian societies like China [20]. It was estimated that approximate 38% of men and 33% of women aged 35–74 years in China had at least one component of MetS, and 14% of men and 18% of women had three or more components of MetS [20]. During past decades, accumulating evidence has highlighted chronic low-grade inflammation as one of the important mechanisms involving in pathogenesis of obesity and related metabolic disorders such as metabolic syndrome, type 2 diabetes and cardiovascular diseases [21]. However, evidence is scarce about the relationships between amylin, inflammatory status and metabolic diseases. Therefore, we aimed to investigate the distribution of plasma amylin and its associations with inflammatory markers and MetS in 1011 apparently healthy Chinese adults. We also examined whether the amylin-MetS association is independent of well established risk factors including obesity, inflammatory markers and insulin resistance.

## Materials and Methods

### Ethics statements

The study was approved by the Institutional Review Board of the Institute for Nutritional Sciences, Chinese Academy of Sciences (Permit No. E-2007-01) and a written informed consent was obtained from each participant.

### Study design

The study design and recruitment of participants have been described in detail elsewhere [22]. In brief, the study population was from the Gut Microbiota and Obesity Study, a population-based case-control study among noninstitutionalized residents aged 35–54 years in Shanghai, China. Two urban districts (Luwan and Zhabei) were chosen to represent people with high and low socioeconomic status in urban Shanghai. Eligible candidates were apparently healthy adults who have lived in Shanghai for at least 10 years. Five hundred pairs of age- and sex-matched subjects (overweight/obesity) and control subjects (normal-weight) were planned to be recruited. Finally, a total of 1,059 eligible participants were successfully recruited from November 2007 to January 2008. Normal weight and overweight/obesity were defined as 18≤BMI<24.0 kg/m^2^ and BMI≥24.0 kg/m^2^, respectively, according to the recommendation by the Working Group on Obesity in China [23]–[25]. The BMI range is 18.0 to 23.9 kg/m^2^ for normal weight and 24.0 to 40.5 kg/m^2^ for overweight/obesity.

Face to face interviews were conducted by trained physicians or public health workers from the local Centers for Disease Control and Prevention, and also from community clinics. Information of demographic variables, health status and behaviors, physical activity and educational attainment were obtained through a standardized questionnaire. Family history of chronic diseases was positive if one of the parents or siblings had coronary heart disease, stroke, or type 2 diabetes. Body weight, height, waist circumference, and blood pressure were measured using a standardized protocol [22].

### Laboratory measurements

Overnight fasting venous blood samples were collected by tubes containing EDTA, centrifuged at 4°C and stored at −80°C until laboratory analyses. Laboratory assays for total cholesterol, high-density lipoprotein (HDL) cholesterol, low-density lipoprotein (LDL) cholesterol, triglycerides, glucose, C-reactive protein (CRP), interleukin 6 (IL-6), and insulin were described previously [22]. The insulin resistance index (homeostasis model assessment of insulin resistance, HOMA-IR) was calculated according to updated homeostasis model assessment methods (http://www.dtu.ox.ac.uk/). Amylin concentrations were measured by a monoclonal antibody-based sandwich immunoassay (Human amylin total ELISA kit, Millipore, Billerica, MA). The assay has a sensitivity of 1 pmol/l with measurable concentrations within 1–100 pmol/l. All procedures followed the manufacture's instructions with two quality controls in expected ranges for each assay. The inter assay-CV was 10% for one of quality controls and was 7.3% for the other one. Every tenth sample was duplicated on the same plate and the average intra-assay CV for amylin was 7.6%. Forty-eight persons with limited plasma samples were excluded and data from 1011 (527 overweight/obese and 484 normal-weight) participants were available for amylin analyses.

### Definition of Mets

MetS was defined based on the updated National Cholesterol Education Program Adult Treatment Panel III criteria for Asian-Americans [17], including at least three of the following components: 1) waist circumference ≥90 cm in men or ≥80 cm in women; 2) triglycerides ≥1.7 mmol/l; 3) HDL cholesterol <1.03 mmol/l in men, or <1.30 mmol/l in women; 4) blood pressure ≥130/85 mm Hg, or current use of antihypertensive medications; and 5) fasting plasma glucose ≥5.6 mmol/l.

### Statistical analyses

Normally distributed variables were expressed as mean ± standard deviation (SD), while variables with a skewed distribution, including amylin, insulin, HOMA-IR, amylin/insulin ratio, triglycerides, CRP and IL-6, were reported as geometric mean (95% confidence interval). Amylin was log transformed to approximate normality before statistical analyses. Categorical variables were represented by frequency and percentage. Analysis of covariance was used to calculate amylin concentrations according to sex, age and BMI. Continuous characteristics across amylin quartiles were compared using analysis of covariance, whereas categorical characteristics across amylin quartiles were compared using logistic regression model. Medians of each amylin quartile were included as an independent variable for trend test. Spearman partial correlation coefficients of amylin with metabolic parameters and inflammatory markers were calculated in the whole sample and subgroups. Multivariate logistic regression models were applied to evaluate the adjusted odds ratios (ORs) for MetS and its components according to the amylin quartiles. Potential confounders were carefully controlled, including age, sex, lifestyle factors, educational attainment, family history of chronic diseases, and inflammatory markers. Potential interactions between BMI, inflammatory markers, insulin, HOMA-IR and amylin were also examined. All statistical analyses were performed with Stata 9.2. (College Station, TX, USA) and considered statistically significant when 2-sided *P*<0.05.

## Results

### Distribution of amylin concentrations and characteristics of participants

As shown in Table 1, geometric means of amylin were significantly higher in older (aged ≥45 years) and overweight/obese participants than their younger (6.85 vs. 6.57 pmol/l, *P* = 0.030) and normal weight counterparts (7.01 vs. 6.46 pmol/l, *P*<0.001). When participants were characterized according to amylin quartiles (Table 2), persons in the higher amylin quartile had significantly higher levels of BMI, waist circumference, blood pressure, fasting glucose, insulin, HOMA-IR, amylin/insulin ratio, total and LDL cholesterol, triglycerides, but lower HDL cholesterol concentrations (all *P*<0.001) compared with those in the lower quartile. Moreover, plasma CRP and IL-6 also elevated along with increased amylin quartiles (both *P*<0.001). We also characterized the participants according to BMI quartiles, and found that plasma amylin concentrations increased gradually according to the quartile of BMI, the geometric means (95% confidence interval [CI]) is 6.21 (5.99–6.44), 6.65(6.40–6.90), 6.84 (6.59–7.10) and 7.20 (6.96–7.46) pmol/l, respectively (*P*<0.001 for trend).

**Table 1:** Amylin concentrations according to sex, age and obesity status (pmol/l)^a^.

	*n*	Geometric mean(95% confidence interval)	*P* value
Sex^b^			0.121
Male	370	6.85 (6.64–7.06)	
Female	641	6.64 (6.49–6.80)	
Age^c^			0.030
<45 years	395	6.57 (6.38–6.77)	
≥45 years	616	6.85 (6.69–7.02)	
Obesity status^d^			<0.001
Normal weight	484	6.46 (6.29–6.64)	
Overweight/Obese	527	7.01 (6.83–7.19)	

a Geometric means of amylin concentrations were compared using general linear models (*n* = 1011).

b Adjustment for BMI and age.

c Adjustment for sex and BMI.

d Adjustment for age and sex.

**Table 2:** Characteristics of participants according to amylin quartiles (*n* = 1011)^a^.

Variables	Q1	Q2	Q3	Q4	*P* for trend
*n* (case/control)	252 (101/151)	253 (129/124)	253 (132/121)	253 (165/88)	
Amylin (pmol/l)	4.71 (4.61–4.82)	6.24 (6.20–6.27)	7.21 (7.18–7.25)	9.59 (9.28–9.90)	<0.001
Age (years)^b^	45.2 (5.5)	45.5 (5.5)	46.0 (5.5)	46.8 (5.1)	<0.001
Male, *n* (%)^b^	94 (37.3)	83 (32.8)	88 (34.8)	105 (41.5)	0.242
Education, *n* (%)					0.994
0–9 years	68 (27.0)	67 (26.5)	73 (28.9)	68 (26.9)	
10–12 years	129 (51.2)	129 (51.0)	129 (51.0)	137 (54.2)	
≥13 years	55 (21.8)	57 (22.5)	51 (20.2)	48 (19.0)	
Current smoker, *n* (%)	59 (23.4)	54 (21.3)	57 (22.5)	74 (29.3)	0.271
Alcohol drinker, *n* (%)	111 (44.1)	74 (29.3)	83 (32.8)	96 (37.9)	0.083
Physical activity, *n* (%)					0.471
Low	13 (5.2)	28 (11.1)	23 (9.1)	32 (12.7)	
Moderate	158 (62.7)	147 (58.1)	145 (57.3)	136 (53.8)	
High	81 (32.1)	78 (30.8)	85 (33.6)	85 (33.6)	
Family history of coronary heart disease, stroke and diabetes, *n* (%)	103 (40.9)	98 (38.7)	101 (39.9)	101 (39.9)	0.693
Metabolic syndrome, *n* (%)	65 (25.8)	83 (32.8)	119 (47.0)	145 (57.3)	<0.001
BMI (kg/m^2^)	23.4 (3.7)	24.2 (3.8)	24.9 (4.2)	25.8 (4.1)	<0.001
Waist circumference (cm)	81.5 (10.5)	82.9 (10.3)	85.3 (11.1)	89.0 (11.2)	<0.001
SBP (mm Hg)	121.7 (15.5)	122.1 (16.0)	126.7 (18.9)	128.1 (18.7)	<0.001
DBP (mm Hg)	76.9 (10.9)	78.2 (11.3)	80.5 (12.0)	81.7 (11.6)	<0.001
Fasting glucose (mmol/l)	5.8 (1.0)	6.0 (1.5)	6.1 (1.3)	6.3 (1.4)	<0.001
Insulin (µU/ml)	7.46 (7.03–7.92)	8.43 (7.88–9.01)	9.52 (8.97–10.11)	11.80 (11.05–12.61)	<0.001
HOMA-IR	0.87 (0.82–0.92)	0.98 (0.92–1.05)	1.11 (1.05–1.18)	1.38 (1.29–1.48)	<0.001
Amylin/insulin	0.09 (0.09–0.10)	0.11 (0.10–0.11)	0.11 (0.10–0.12)	0.12 (0.11–0.13)	<0.001
Total cholesterol (mmol/l)	5.0 (1.0)	5.1 (1.2)	5.4 (1.2)	5.4 (1.1)	<0.001
LDL cholesterol (mmol/l)	3.1 (0.8)	3.2 (1.0)	3.4 (1.0)	3.4 (0.9)	<0.001
HDL cholesterol (mmol/l)	1.5 (0.4)	1.4 (0.4)	1.4 (0.4)	1.3 (0.4)	<0.001
Triglycerides (mmol/l)	1.01 (0.95–1.07)	1.10 (1.03–1.18)	1.36 (1.26–1.46)	1.70 (1.57–1.84)	<0.001
CRP (mg/l)	0.74 (0.66–0.82)	0.83 (0.74–0.94)	1.00 (0.88–1.13)	1.17 (1.04–1.31)	<0.001
IL-6 (pg/ml)	1.33 (1.23–1.43)	1.27 (1.17–1.37)	1.45 (1.34–1.56)	1.63 (1.50–1.76)	<0.001

a Data are arithmetic mean (SD) or geometric mean (95% confidence interval) if not specified. Percentages may not sum to 100 because of rounding. *P* for trend was calculated after adjustment for age and sex.

b Not adjusted for itself.

### Correlations of amylin concentrations with metabolic parameters and inflammatory markers

Amylin was positively correlated with BMI, waist circumference, blood pressure, fasting glucose, insulin, HOMA-IR, total cholesterol, LDL-cholesterol, triglycerides, inflammatory markers of CRP and IL-6, while, negatively correlated with plasma HDL-cholesterol (Table 3, all *P*<0.001), after adjustment for age and sex. Among all the metabolic parameters, amylin showed the strongest correlation with insulin and HOMA-IR. In stratified analyses, the correlations were generally stronger in overweight/obese and male participants than in their normal-weight and female peers.

**Table 3:** Partial spearman correlation coefficients of amylin with metabolic parameters and inflammatory markers.

	Total^a^(*n* = 1011)		Normal weight^a^(*n* = 484)		Overweight/obese^a^(*n* = 527)		Male^b^(*n* = 370)		Female^b^(*n* = 641)	
	*r*	*P* value	*r*	*P* value	*r*	*P* value	*r*	*P* value	*r*	*P* value
BMI	0.21	<0.001	0.12	0.007	0.18	<0.001	0.29	<0.001	0.17	<0.001
Waist circumference	0.24	<0.001	0.18	<0.001	0.20	<0.001	0.34	<0.001	0.18	<0.001
SBP	0.13	<0.001	0.05	0.229	0.08	0.062	0.19	<0.001	0.08	0.044
DBP	0.14	<0.001	0.07	0.131	0.09	0.042	0.19	<0.001	0.11	0.007
Fasting glucose	0.15	<0.001	0.10	0.027	0.16	<0.001	0.17	0.001	0.14	<0.001
Insulin	0.33	<0.001	0.20	<0.001	0.38	<0.001	0.39	<0.001	0.28	<0.001
HOMA-IR	0.33	<0.001	0.21	<0.001	0.38	<0.001	0.40	<0.001	0.29	<0.001
Total cholesterol	0.12	<0.001	0.07	0.108	0.13	0.004	0.18	<0.001	0.06	0.159
LDL-cholesterol	0.12	<0.001	0.09	0.040	0.10	0.027	0.16	0.002	0.08	0.045
HDL-cholesterol	−0.18	<0.001	−0.08	0.080	−0.18	<0.001	−0.25	<0.001	−0.13	0.001
Triglycerides	0.31	<0.001	0.17	<0.001	0.35	<0.001	0.39	<0.001	0.24	<0.001
CRP	0.18	<0.001	0.10	0.027	0.14	0.002	0.19	<0.001	0.16	<0.001
IL-6	0.11	<0.001	0.06	0.159	0.08	0.054	0.11	0.034	0.11	0.006

a Correlation coefficients were calculated after adjustment for age and sex.

b Correlation coefficients were calculated after adjustment for age only.

### Associations of amylin with metabolic syndrome and its components

**Figure 1:**
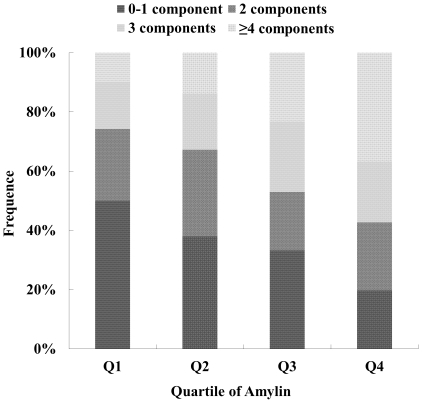
The severity of MetS according to amylin quartiles. *P*<0.001 for trend.

The prevalence of MetS progressively increased from 25.8% to 57.3% across amylin quartiles (Table 4). The severity of MetS, indicated as ≤1, 2, 3 and ≥4 components, also increased gradually across amylin quartiles (Figure 1). Table 4 (model 2) showed that, compared with the lowest quartile of amylin, the ORs in the highest quartile were 3.71 (95% CI: 2.53–5.46) for MetS, 2.72 (1.88–3.93) for central obesity, 1.66 (1.14–2.41) for elevated blood pressure, 1.88 (1.28–2.76) for hyperglycemia, 5.55 (3.58–8.61) for hypertriglyceridemia, and 2.56 (1.75–3.76) for low HDL cholesterol (*P*<0.001 for trend for the overall risk of MetS and most of the features except elevated blood pressure with *P* for trend = 0.002). Further adjustment for BMI and inflammatory markers (model 4) only slightly reduced the magnitude of the association of amylin with MetS (with an OR of 2.41, 95% CI: 1.46–3.97). Although further adjustment of insulin based on model 2 decrease the magnitude of the amylin-MetS association (with an OR of 1.85, 95% CI: 1.21–2.85), the association of amylin with MetS is still statistically significant (*P* = 0.001 for trend). These results suggest that amylin is associated with MetS independent of obesity, inflammatory markers or insulin.

**Table 4:** Odds ratios and 95% confidence interval for MetS and its individual components according to amylin quartiles (*n* = 1011).

Odds Ratios (95% CI)					
	Q1(<5.70)	Q2(5.70–6.75)	Q3(6.75–7.83)	Q4(≥7.83)	*P* for trend
Metabolic syndrome	65/252	83/253	119/253	145/253	
Model 1	1	1.42 (0.96–2.09)	2.54 (1.74–3.71)	3.66 (2.51–5.36)	<0.001
Model 2	1	1.43 (0.96–2.12)	2.54 (1.73–3.72)	3.71 (2.53–5.46)	<0.001
Model 3	1	1.23 (0.75–2.00)	2.33 (1.43–3.80)	2.53 (1.55–4.14)	<0.001
Model 4	1	1.25 (0.76–2.06)	2.22 (1.35–3.65)	2.41 (1.46–3.97)	<0.001
Model 5	1	1.19 (0.77–1.82)	1.86 (1.22–2.82)	1.85 (1.21–2.85)	0.001
Model 6	1	1.16 (0.75–1.79)	1.81 (1.18–2.75)	1.75 (1.13–2.70)	0.003
Central obesity	98/252	125/253	133/253	162/253	
Model 1	1	1.51 (1.06–2.16)	1.71 (1.20–2.44)	2.75 (1.91–3.95)	<0.001
Model 2	1	1.47 (1.03–2.11)	1.66 (1.16–2.38)	2.72 (1.88–3.93)	<0.001
Model 3	1	1.07 (0.55–2.11)	1.04 (0.53–2.04)	1.29 (0.64–2.58)	0.505
Model 4	1	1.11 (0.56–2.21)	0.99 (0.50–1.96)	1.24 (0.61–2.51)	0.617
Model 5	1	1.26 (0.85–1.86)	1.16 (0.78–1.71)	1.39 (0.92–2.10)	0.160
Model 6	1	1.24 (0.84–1.84)	1.13 (0.77–1.68)	1.35 (0.89–2.04)	0.216
Elevated blood pressure	90/252	87/253	111/253	125/253	
Model 1	1	0.96 (0.66–1.40)	1.40 (0.96–2.02)	1.59 (1.10–2.30)	0.004
Model 2	1	0.99 (0.67–1.45)	1.43 (0.98–2.08)	1.66 (1.14–2.41)	0.002
Model 3	1	0.84 (0.56–1.27)	1.11 (0.74–1.67)	1.04 (0.69–1.57)	0.598
Model 4	1	0.85 (0.56–1.29)	1.07 (0.71–1.62)	1.00 (0.66–1.51)	0.768
Model 5	1	0.87 (0.59–1.30)	1.15 (0.78–1.69)	1.07 (0.72–1.60)	0.515
Model 6	1	0.86 (0.58–1.28)	1.13 (0.76–1.66)	1.03 (0.69–1.55)	0.624
Hyperglycemia	144/252	143/253	165/253	184/253	
Model 1	1	0.95 (0.67–1.36)	1.36 (0.94–1.95)	1.89 (1.29–2.75)	<0.001
Model 2	1	0.96 (0.67–1.38)	1.37 (0.95–1.97)	1.88 (1.28–2.76)	<0.001
Model 3	1	0.90 (0.62–1.29)	1.21 (0.84–1.77)	1.54 (1.04–2.28)	0.014
Model 4	1	0.86 (0.60–1.25)	1.16 (0.80–1.70)	1.49 (1.00–2.22)	0.022
Model 5	1	0.92 (0.64–1.32)	1.23 (0.85–1.79)	1.54 (1.03–2.29)	0.017
Model 6	1	0.89 (0.62–1.28)	1.16 (0.80–1.69)	1.38 (0.92–2.07)	0.067
Hypertriglyceridemia	38/252	57/253	85/253	124/253	
Model 1	1	1.74 (1.09–2.76)	3.01 (1.93–4.68)	5.42 (3.51–8.37)	<0.001
Model 2	1	1.77 (1.11–2.82)	3.07 (1.96–4.79)	5.55 (3.58–8.61)	<0.001
Model 3	1	1.60 (0.99–2.59)	2.53 (1.60–4.02)	4.15 (2.64–6.54)	<0.001
Model 4	1	1.61 (0.99–2.61)	2.45 (1.53–3.91)	4.07 (2.57–6.45)	<0.001
Model 5	1	1.54 (0.96–2.49)	2.40 (1.52–3.80)	3.48 (2.20–5.51)	<0.001
Model 6	1	1.52 (0.94–2.46)	2.34 (1.47–3.71)	3.32 (2.09–5.28)	<0.001
Low HDL cholesterol	69/252	91/253	92/253	117/253	
Model 1	1	1.51 (1.03–2.21)	1.58 (1.08–2.31)	2.51 (1.72–3.67)	<0.001
Model 2	1	1.53 (1.04–2.25)	1.58 (1.07–2.32)	2.56 (1.75–3.76)	<0.001
Model 3	1	1.39 (0.93–2.07)	1.29 (0.87–1.93)	1.88 (1.26–2.81)	0.003
Model 4	1	1.42 (0.95–2.12)	1.30 (0.87–1.95)	1.87 (1.25–2.79)	0.004
Model 5	1	1.38 (0.92–2.05)	1.24 (0.83–1.86)	1.63 (1.08–2.45)	0.034
Model 6	1	1.38 (0.93–2.06)	1.26 (0.85–1.87)	1.67 (1.11–2.52)	0.024

Model 1, adjusted for age and sex;

Model 2, further adjusted for smoking, alcohol drinking, physical activity, educational attainment and family history of chronic diseases based on model 1;

Model 3, further adjusted for BMI based on model 2;

Model 4, further adjusted for inflammatory markers (CRP and IL-6) based on model 3.

Model 5, further adjusted for insulin based on model 2;

Model 6, further adjusted for HOMA-IR based on model 2.

**Figure 2:**
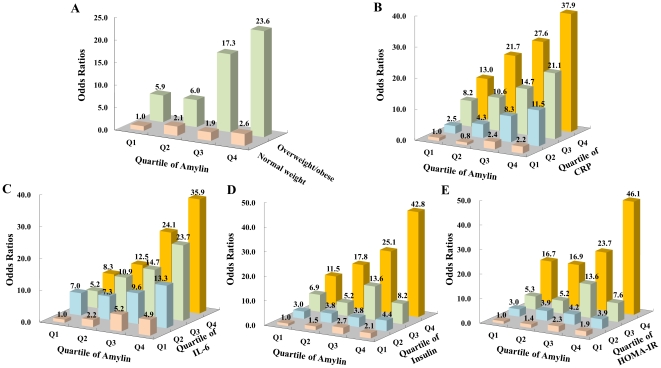
Adjusted odds ratios for MetS according to amylin quartiles, obesity status (A), and quartiles of CRP (B), IL-6 (C), insulin (D), HOMA-IR (E). Adjusted for age, sex, smoking, alcohol drinking, physical activity, educational attainment and family history of chronic diseases. A: Modified MetS was defined as having two or more components of the MetS without central obesity. Normal weight: 18≤BMI<24 kg/m^2^; overweight/obese: BMI≥24.0 kg/m^2^. *P* = 0.105 for interaction of amylin and BMI, *P* = 0.443 for interaction of amylin and CRP; *P* = 0.448 for interaction of amylin and IL-6; *P* = 0.375 for interaction of amylin and insulin; *P* = 0.397 for interaction of amylin and HOMA-IR.

In joint classification analyses, rising amylin was associated with approximately 2–5 fold higher risk of MetS in the individuals with normal-weight (OR = 2.6 [95% CI: 1.4–4.7] for Q4 *vs.* OR = 1 [reference group], Figure 2A) and low inflammatory status indicated as in the lowest quartile of CRP (OR = 2.2 [95% CI: 0.7–7.4] for Q4 *vs.* OR = 1.0 for Q1, Figure 2B), IL-6 (OR = 4.9 [95% CI: 1.6–15.6] for Q4 *vs.* OR = 1.0 for Q1, Figure 2C), insulin (OR = 2.1 [95% CI: 0.7–6.2] for Q4 *vs.* OR = 1.0 for Q1, Fig. 2D), and HOMA-IR (OR = 1.9 [95% CI: 0.6–6.0] for Q4 *vs.* OR = 1.0 for Q1 Fig. 2E). However, the risk of MetS was more pronounced among the participants who were overweight/obese (OR = 23.6 [95% CI: 12.9–43.1] for Q4 *vs.* OR = 5.9 [95% CI: 3.3–10.5] for Q1) or in the highest quartile of plasma CRP (OR = 37.9 [95% CI: 14.1–101.8] for Q4 *vs.* OR = 13.0 [95% CI: 4.6–36.5] for Q1), IL-6 (OR = 35.9 [95% CI: 12.5–102.9] for Q4 *vs.* OR = 8.3 [95% CI: 2.9–23.8] for Q1), insulin (OR = 42.8 [95% CI: 18.7–97.6] for Q4 *vs.* OR = 11.5 [95% CI: 4.2–31.9] for Q1) and HOMA-IR (OR = 46.1 [95% CI: 20.0–106.1] for Q4 *vs.* OR = 16.7 [95% CI: 5.9–47.3] for Q1) in combination with elevated circulating amylin. No significant interactions were observed between amylin, obesity, inflammatory status and insulin resistance on the risk of MetS (*P*>0.05 for all interaction tests).

## Discussion

In this study, we found that elevated amylin concentrations were significantly correlated with inflammatory status and unfavorable metabolic traits. The strong positive association between amylin and the risk of MetS was independent of the well established risk factors, including obesity, inflammatory markers (CRP and IL-6) and insulin resistance in apparently healthy Chinese population.

Few studies have examined the distribution of circulating amylin in a large population. Previously, Reinehr *et al* reported that serum amylin of 37 obese children was significantly higher than that in 16 health lean children, but was not correlated with age, sex and BMI [13]. Obese persons with/without impaired glucose tolerance were found to have increased circulating amylin [13], [14], [26], [27] and obese individuals also tended to have higher amylin response toward glucose load compared with non-obese individuals [27]. With a relatively large sample, our data showed that increased amylin concentrations across quartiles were associated with not only advanced age and higher BMI, but also unfavorable metabolic and inflammatory profiles (Table 2). In general, the correlations were stronger in overweight/obese participants than their normal-weight counterparts, particularly in the case of insulin, HOMA-IR and triglycerides. However, the mechanistic linkage between elevated amylin levels and obesity related metabolic disorders has not been fully understood thus far. Indeed, data from a weight loss intervention in obese children suggested that changing of amylin levels was correlated with altered insulin rather than change of BMI or percentage of body fat [13].

One of the major findings of our current study is that we discovered a strong association between plasma amylin and the risk of MetS. Accumulating evidence, mainly from cellular and animal studies, suggested that amylin plays important roles in regulating food intake, insulin action and energy homeostasis, as well as glucose and lipid metabolisms. Moreover, adverse effects of increased amylin levels on metabolic abnormalities such as insulin resistance or type 2 diabetes, have been indicated by some of human studies [13], [14]. However, little is known whether high circulating amylin is an independent risk factor for MetS, a preclinical condition associated with a 2-fold increase in risk of CVD, CVD mortality, and stroke [18]. In this study, we found that elevated amylin *per se* is associated with higher risk of MetS even in normal weight persons, despite the fact that the MetS risk was pronounced in overweight/obese participants (Figure 2A). Obesity is a well established risk factor of MetS [28]–[30]. Adjustments for BMI in combining other potential confounders, however, yielded only a minor reduction on the risk of MetS across amylin quartiles (Table 4). Thus, the amylin-MetS association in the current study apparently could not mainly be attributed to obesity. Hyperinsulinemia associated with the insulin resistance of obesity is also a defining marker of MetS. Our present study showed that amylin and insulin were both elevated in obesity and insulin resistance, which is consistent with previous reports [13], [14], [26], [27]. In addition, we found that amylin was associated with MetS independent of hyperinsulinemia/insulin resistance. Although the secretion of amylin and insulin is generally parallel under physiological conditions, these two hormones are differently regulated under certain circumstances. High glucose increases the relative amount of amylin to insulin secretion from islets isolated from dexamethasone- or glucose-treated rats in comparison with islets isolated from fed and fasted rats [31]. Increased storage and secretion of amylin relative to insulin has been reported in spontaneously diabetic GK rats [32]. We found that participants in the higher amylin quartile had higher amylin/insulin ratio and higher risk of MetS, supporting the involvement of amylin in MetS independent of insulin.

Another noteworthy finding in our study was that we observed a significantly positive correlation between amylin and inflammatory markers (CRP and IL-6) (Table 3). Low-grade inflammation is one of the widely accepted key mechanisms related to obesity, insulin resistance, type 2 diabetes and cardiovascular diseases, the conditions often coexisted with hyperamylinemia. However, direct evidence was scarce regarding the relationship between amylin and inflammation. In our recent *in vitro* study, we observed that tumor necrosis factor- α (TNF-α), a proinflammatory cytokine elevated in obesity and insulin resistance, could induce amylin gene expression in murine pancreatic β-cells and islets, and also could activate human amylin promoter through multiple signaling pathways [33]. Masters et al. [34] recently reported that amylin triggered the NLRP3 inflammasome and generated mature IL-1β, an important inflammatory mediator in type 2 diabetes, in macrophages. Mice transgenic for human amylin had more IL-1β in pancreatic islets, which localized together with amyloid and macrophages. While it is still waiting to be verified whether the findings from the in vitro and animal studies are biologically relevant to humans, our data, however, showed that elevated amylin was significantly correlated with increased plasma CRP and IL-6. Interestingly, like in the case of obesity and insulin resistance, the effect of CRP and IL-6 on the amylin-MetS association was rather minor (Table 4). Together, this data again suggests that amylin might independently promote metabolic disorders through the mechanism(s) other than inflammatory signaling pathway.

In the present study, we also documented that increased circulating amylin was significantly associated with the severity (Figure 1) and each component of MetS (Table 4). Interestingly, the association between amylin and hypertriglyceridemia was particularly strong and also independent of BMI, a well recognized risk factor for hypertriglyceridemia [35], whereas the associations between amylin and the rest of the MetS components were largely explained by BMI (Table 4). Previously, a positive correlation between amylin and triglycerides was reported based on a study of 53 children [13]. However, it remains controversial whether elevated amylin directly or indirectly induce dyslipidemia or *vice versa*. For instance, Smith and coworkers reported that a bolus injection or infusion with amylin significantly raised total plasma triglyceride levels and reduced clearance of TG-rich lipoproteins by about 45% [36]. In another study, Ye *et al* showed that amylin infusion in rats increased not only circulating levels of non-esterified fatty acids and glycerol, but also hepatic triglyceride content [37]. On the other hand, we found that acute treatment with dietary fatty acids could enhance amylin expression and secretion in murine pancreatic β cells [4], as well as stimulate human amylin promoter activation. Furthermore, acutely fed mice with high-lipid contained diet could raise plasma levels of fatty acid, amylin and insulin in a temporal manner, implicating fatty acid might play a critical role in inducing amylin release [4]. The discrepancies might be due to inter-species variations of animal models, different protocols and doses used among studies. Nonetheless, long-term studies, especially human studies, are merited to prove the observations from acute animal studies and also to identify mechanism(s) linking between hyperamylinemia and dyslipidemia.

To our knowledge, this is the first study to evaluate the association between amylin levels and the risk of MetS in a relatively large-scale population. Admittedly, due to the cross-sectional nature of current study, we can not establish a causal association of amylin with MetS risk and its components. We have made efforts to eliminate possible effects of most potential confounders by employing strict exclusion criteria and recruiting a relatively large sample of apparently healthy adults with both sexes. A randomized study with a sufficiently large sample will further decrease the potential role of residual confounding. Certainly, our results should be examined in longitudinal studies to establish the causal relationship between amylin and metabolic syndrome. Whether increased amylin could serve as a useful biomarker or intervention target in clinical settings for predicting and controlling metabolic diseases should be determined prospectively among different populations.

In summary, our findings suggest that elevated circulating amylin is strongly associated with MetS, independent of established risk factors including obesity, inflammatory markers and insulin resistance in apparently healthy Chinese. Our results also provide novel insights into the potential role(s) of amylin in the development of metabolic diseases.
